# A Morphometric Study of the Retromolar Fossa and the Incidence of the Retromolar Foramen in Dry Human Mandibles

**DOI:** 10.7759/cureus.66050

**Published:** 2024-08-03

**Authors:** Dwiz Dwivedi, Noor Us Saba, Pratibha Shakya, Heena Singh, Navneet Chauhan

**Affiliations:** 1 College of Medicine, King George's Medical University, Lucknow, IND; 2 Anatomy, King George's Medical University, Lucknow, IND

**Keywords:** retromolar canal, retromolar trigone, retromolar foramen, retromolar fossa, mandible

## Abstract

Introduction: The mandible is the largest and strongest facial bone which plays a crucial role for various surgeries and diagnostic imaging. The retromolar fossa, located behind the third molar socket on each side, was observed for morphometry of anterior, medial, and posterior borders. The present study aimed to assess the retromolar fossa and the presence of retromolar foramen.

Methods: This cross-sectional study was conducted on 30 dry mandibles of adult humans of unknown sex; morphometry of retromolar foramen was done using three parameters: anteriorly, laterally, and posteroinferiorly. Dimensions of the retromolar trigone were seen with notable differences between the right and left sides.

Results: Dimensions of the anterior border had a mean of 12.34±1.175 mm on the right side and 12.56±1.46 mm on the left side. The mean of the medial border of the trigone on the right side was 20.23±2.84 mm and on the left side was 21.48±2.57 mm. The lateral border had the mean value of 18.33±3.56 mm and 19.21±3.93 mm on the right and left sides respectively. The P-value of the medial border was found to be statistically significant (P=0.02). Retromolar foramen was observed in 60% of mandibles; six were unilateral and 12 were bilateral. These foramina were closer to the anterior border of the retromolar trigone as compared to the anterior border of the ramus and lingula of the mandibles.

Conclusions: The awareness of these findings is crucial for the prevention of complications, such as neurovascular damage, during surgeries in this region. This knowledge is particularly relevant in addressing oral pathologies and extracting third molars, contributing to improved surgical outcomes and patient safety.

## Introduction

The mandible is the largest and strongest bone of the face. It consists of two ramus and a body. The upper border of the body has a "U" shaped alveolar arch to embed the teeth of the lower jaw in their sockets [1]. Retromolar fossae are two triangular areas present behind the socket of the third molar on each side. These are small depressions, having a temporal crest medially, a continuation of the anterior border of the ramus of the mandible laterally, and the posterior margin of the third molar anteriorly [2].

Retromolar foramina and their continuation into the retromolar canal can also be seen in these fossae. The presence of foramina and canals in retromolar trigone has clinically important neurovascular passages [3,4]. Knowledge of the retromolar fossa and the presence of retromolar foramen is important for surgeons in orthognathic surgeries, extraction of the third molar, periodontal wedge procedures, sagittal split osteotomy as well as for radiologists. It is a potential space to accommodate various neoplasms and oral cancers [5,6]. The present study aimed to assess the retromolar fossa and the presence of retromolar foramen.

## Materials and methods

This cross-sectional study was conducted on 30 dry mandibles of adult humans of unknown sex in the Department of Anatomy, King George’s Medical University, Lucknow, India. This study was approved by the Institutional Ethics Committee with ref. code: XIX-PGTSC-IIB-IMR-S/P1 dated 05/08/2023. Mandibles with sockets of third molar teeth were selected, whereas distorted and damaged bones were excluded.

**Figure 1:**
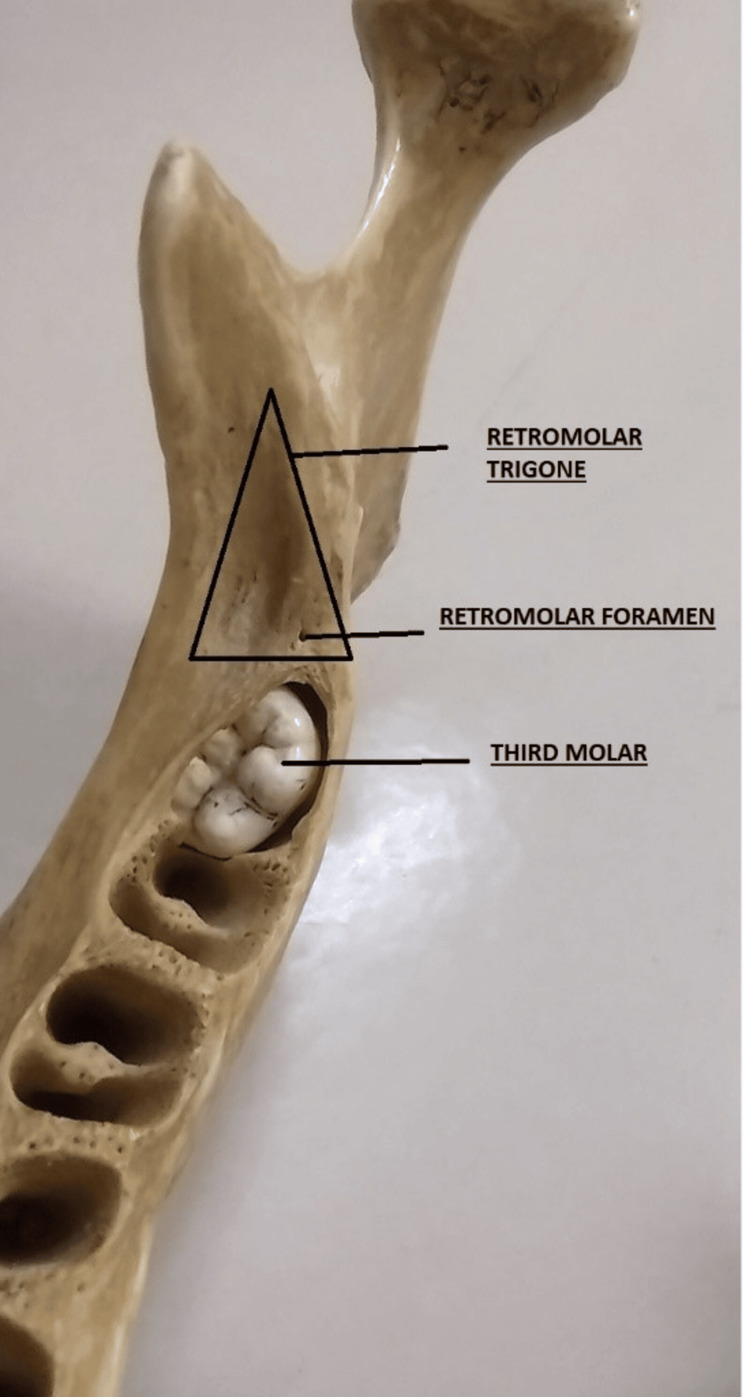
Retromolar trigone showing its medial border, lateral border, and anterior border.

Morphometry of the retromolar fossa was done bilaterally on the mandible with the help of the vernier caliper. The anterior, medial, and lateral borders of the retromolar fossa were measured in millimetres (mm) to make a trigone just behind the third molar tooth (Figure 1).

**Figure 2:**
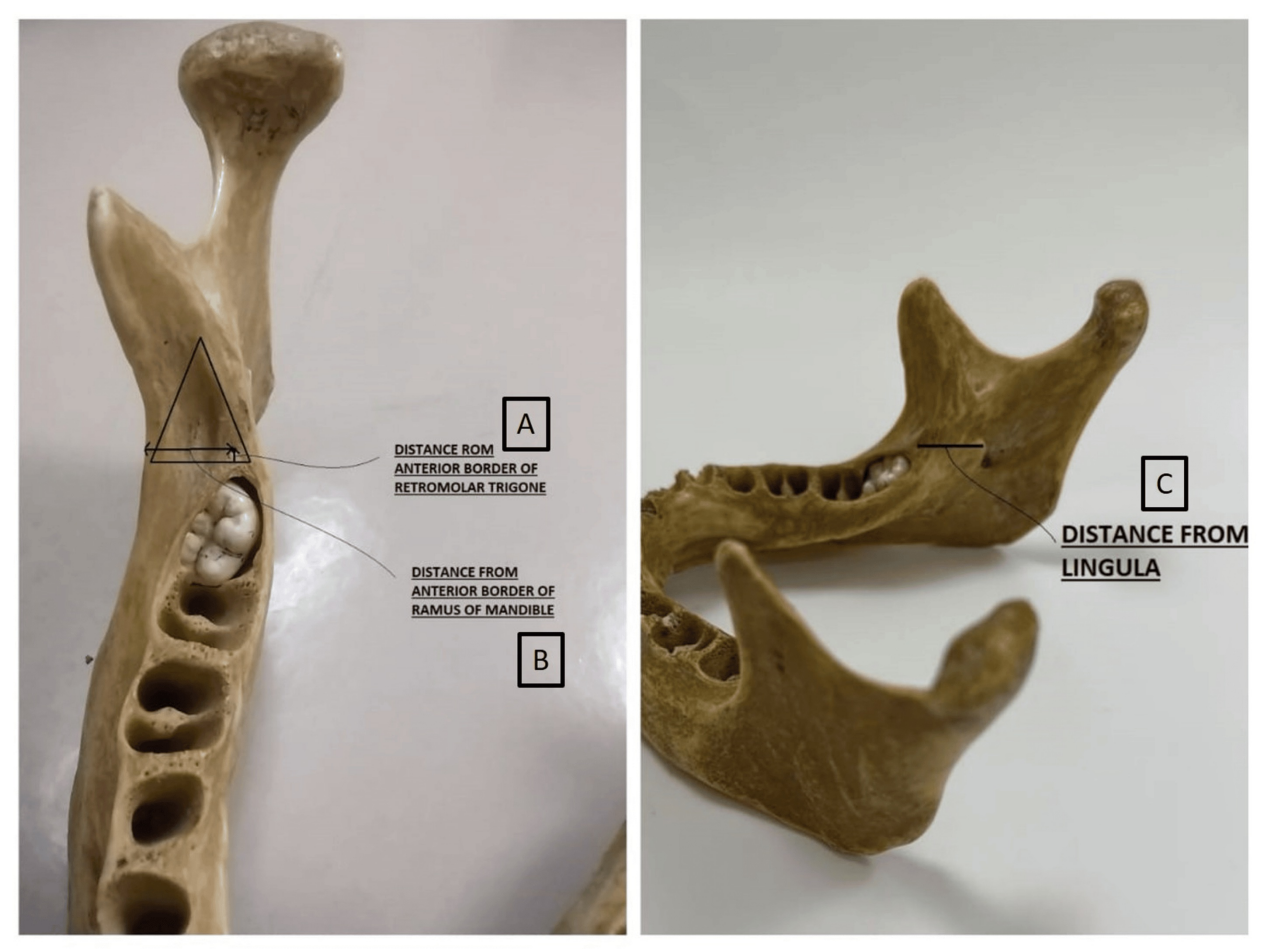
Measurements taken for the location of the retromolar foramen: (A) from the anterior border of the retromolar trigone; (B) from the anterior border of the ramus of the mandible; (C) from the lingula of the mandible on the same side as the retromolar foramen.

The location of the retromolar foramen was measured in a distance of millimetres (mm) from the anterior border, from the ramus of the mandible laterally, and from the lingula posteroinferiorly (Figure 2).

The study was analysed by using single tail-paired t-test in an Excel file (Microsoft® Corp., Redmond, WA, USA). Mean, standard deviation (SD) and P-value were calculated.

## Results

Measurements of retromolar trigone (Table 1) and retromolar foramen were taken and tabulated for both the right and left sides of the mandibles (Table 2).

**Table 1:** Dimensions of retromolar trigone (readings in mm)

	Left Side			Right Side		
Mandible	Anterior border	Medial border	Lateral border	Anterior border	Medial border	Lateral border
1	13.03	21.1	21.05	13.05	18.85	13.4
2	12.4	17.15	16.45	12.2	19.4	15.3
3	11.05	23.15	26.05	11.1	20.35	19.15
4	11.1	26.45	24.2	11.2	23.2	27.5
5	10.1	18.2	18.5	10.1	10.3	19.8
6	12.3	21.1	19.1	13.3	25.15	22.7
7	14.5	25.1	21.5	11.1	20.5	18.75
8	14.7	20.9	21.8	12.8	20.1	18.3
9	11.1	17.35	11.1	12.8	18.1	16.1
10	14.8	22.95	22.2	12.2	18.9	17.4
11	11.6	19.1	16.75	11.7	19.45	11.05
12	13.35	21.4	18.05	13.25	20.05	17.2
13	13.05	19.05	21.1	12.7	19.1	15.9
14	11.4	19.05	18.55	11.65	19.35	16.1
15	14.4	24.1	22.45	13.25	22.15	21.2
16	14.3	23.4	24.05	15.05	24.05	25.2
17	9.55	20.35	21.05	11.05	20.65	18.1
18	14.1	24.5	21.75	14.65	23.6	19.6
19	14.6	20.8	19.7	13.8	19.55	17.05
20	12.2	21.85	20.75	11.7	20.4	18.05
21	13.6	22.05	22.8	11.45	22.4	21.05
22	14.2	22.85	21.7	13.45	20.55	20.25
23	13.65	24.8	23.9	14.65	26.1	23.1
24	12.2	17.45	21.5	12.3	21.23	26.3
25	11.3	26.15	18.15	11.4	18.4	16.7
26	11.1	19.3	24.3	11.5	18.5	18.5
27	13.2	20.15	13.2	12.5	23.2	16.8
28	10.8	21.4	14.5	11.1	22.75	16.3
29	12.6	24.35	11.25	12.2	17.2	15.5
30	13.3	23.45	12.4	12.8	20.4	17.6

**Table 2:** Measurements of the location of the retromolar foramen (readings in mm)

Left Side				Right Side			
S. no. of bone	Distance from the anterior border	Distance from the anterior border of the ramus	Distance from lingula	S. no. of bone	Distance from the anterior border	Distance from the anterior border of the ramus	Distance from lingula
1	Just at anterior border	9.7	13.65	1	1.35	9.15	13.2
3	7.6	10.7	15.5	3	Just behind anterior border	10.1	21.5
4	7.05	10.1	12.5		Just behind anterior border	11.1	20.1
	5.1	9.35	12.2	4	7.6	10.1	13.5
	5.1	7.35	13.3	5	2.05	9.8	12.05
7	9.8	15.8	9.4	7	10.2	8.85	13.7
8	Just behind anterior border	12.25	15.3	11	1.2	8.1	14.55
	Just at anterior border	12.05	14.65	13	1.9	8.75	13.05
11	1.05	8.45	13.1		6.1	5.1	11.05
12	Just at anterior border	11.1	13.8	15	Just at anterior border	10.5	14.9
	0.5	6.65	13.5	17	Just behind anterior border	9.1	12.3
13	Just at anterior border	10.05	13.5	18	Just at anterior border	12.05	18.5
	5.4	5.05	11.75	23	2.25	10.8	13.05
17	Just behind anterior border	8.25	13.05	25	8.65	6.8	15.7
22	4.45	8.75	14.35	27	3	11	17.75
	6.5	9.2	14.15	29	2.3	12.5	12.4
23	4.05	10.05	15.6	30	8.5	9.35	13.3
	9.85	7.05	15.5	-	-	-	-
25	3.25	10.1	12.5	-	-	-	-
27	2.75	8.9	14.35	-	-	-	-
29	4.5	7.1	13.35	-	-	-	-
30	6.75	11.1	11.5	-	-	-	-

Borders of retromolar trigone

The length of the anterior border of retromolar trigone varies from 10.1 mm to 15.05 mm on the right side (mean=12.34±1.175), and 9.55 mm to 14.80 mm on the left side (mean=12.56±1.46) respectively. Similarly, the length of the medial border of the trigone on the right side was between 10.3 mm and 25.15 mm (mean=20.23±2.84), and 17.15 mm to 26.45 mm (mean=21.48±2.57) on the left side. Lastly, the length of the lateral border varies from 11.05 mm to 27.5 mm on the right side (mean=18.33±3.56), and 11.1 mm to 26.05 mm on the left side (mean=19.21±3.93). The P-value of the medial border was found to be statistically significant (P=0.02) (Table 3).

**Table 3:** Retromolar trigone in the mandibles (readings in mm)

	Right Side (Mean)	Right Side (SD)	Left Side (Mean)	Left Side (SD)	P-Value (Single Side)
Anterior border	12.34526	1.175798	12.56568	1.462238	0.114703
Medial border	20.2298	2.845902	21.47896	2.570257	0.022268
Lateral border	18.33479	3.564504	19.20686	3.928224	0.074621

Retromolar foramen

The occurrence of retromolar foramen was seen in 18 out of 30 mandibles i.e., 60%. They were unilateral in six mandibles and bilateral in 12 mandibles. Unilateral double retromolar foramen was present on the left side in three mandibles and unilateral single retromolar foramen was seen on the right side in the other three mandibles. Three retromolar foramen on the left side were also observed in one of the mandibles having foramen bilaterally. These foramina were generally present near the anterior border of the retromolar trigone i.e., near the third molar socket. They were closer to the anterior border of the ramus in comparison to the lingula (Table 2).

The average distance of the foramen from the anterior border of the trigone was 4.59±3.52 mm on the right side, and 5.23±3.29 mm on the left side. The distance from the anterior border of the ramus was 9.59±1.83 mm on the right side, and 9.50±2.29 mm on the left side. This distance of foramen from the lingula was 14.74±2.99 mm on the right side, and 13.48±1.50 mm on the left side (Table 4).

**Table 4:** Location of the retromolar foramen (readings in mm)

	Left Side (Mean)	Left Side (SD)	Right Side (Mean)	Right Side (SD)
Distance from anterior border	5.23125	3.287853153	4.591666667	3.517111532
Distance from anterior border of ramus	9.504545455	2.28545656	9.597058824	1.829486829
Distance from lingula	13.47727273	1.494890287	14.74117647	2.996520654

## Discussion

Various studies in India [7-9] and around the globe as Japan [10], China [11], Brazil [12], and the USA [13] have been carried out to observe the retromolar trigone and find the incidence of retromolar foramen. A wide range (8-72%) of variation was observed in comparing the findings of different studies. The probable cause of these differences can be related to the development of the mandible, and having many nerve canals in the initial stages of the embryonic period. These multiple nerve channels may or may not disappear in a later stage, leaving a range of presentations from a single canal in the mandible to a combination of numerous small passages taking origin from the chief mandibular canal [5]. Other causes might include the differences in populations in terms of their ethnicity, region, and climatic conditions.

In our study, the size of the trigone was observed in parallel with various other studies. Dimensions of the anterior border were similar to the observations of Potu et al. [9], whereas dimensions of the medial and lateral walls were quite less in the present study. This difference is attributed to the sample difference and regional differences in their study. The P-value in the present study was significant for the dimensions of the medial border of the trigone (Table 3).

The frequency of observation of retromolar foramina was 60% in the present study which was in line with the findings of Kawai et al. [10] and Schejtman et al. [14]. Incidence was very low in the studies of Priya et al., Athavale et al., Potu et al., Galdámes et al. and Sawyer et al. [7-9,12,13] as compared to the current study (Table 5). The retromolar foramen is an anatomical variation consisting of neurovascular supply through the retromandibular canal [15]. Any damage to the supply during surgeries can cause various complications as reported by Singh [16]. Singh reported complete paraesthesia of the buccal mucosa due to injury in the retromolar region during the third molar extraction. Branching of the nerves and vessels from the mandibular canal to exit from the retromandibular foramen might be responsible for incomplete anaesthesia of the retromolar region. The presence of variations in the vasculature in the retromandibular foramen and retromandibular canal may cause incomplete anaesthesia in this region during surgeries [3]. It is important to consider the presence of retromandibular foramen while performing various oral surgeries specifically during third molar extraction and sagittal split osteotomy. Also, the neoplasms of the retromolar region tend to metastasise through the vasculature into the surrounding tissues [4,17].

**Table 5:** Comparison of different studies

Study	Country	Sample Size	Presence of Retromolar Foramen
Present study	India	30	18 (60%)
Schejtman et al. (1967) [14]	Argentina	18	13 (72%)
Sawyer et al. (1991) [13]	USA	234	18 (7.69%)
Priya et al. (2005) [7]	India	157	20 (12.74%)
Kawai et al. (2012) [10]	Japan	46	24 (52.17%)
Galdámes et al. (2008) [12]	Brazil	294	38 (12.92%)
Athavale et al. (2013) [8]	India	71 bones and 10 cadavers	10 (14.08%)
Potu et al. (2014) [9]	India	94	11 (11.70%)
Ren et al. (2023) [11]	China	123	39 (31.70%)

## Conclusions

Awareness of the anatomical variations in the dimensions of the retromolar fossa and the incidence of retromolar foramen helps surgeons and anaesthetists to do various procedures in the oral cavity. The cancer of the trigone as well as the extraction of the third molar requires information regarding its dimensions and the presence of retromolar foramen within the trigone.
